# Time to viral load suppression after enhanced adherence counseling among HIV patients attending antiretroviral therapy in Southwest Ethiopia

**DOI:** 10.1186/s12879-026-12957-9

**Published:** 2026-02-24

**Authors:** Haymanot Berihun, Abel Felege, Daniel Girma, Dereje Tsegaye

**Affiliations:** Department of Public Health, College of Health Sciences, Mattu University, Mattu, Ethiopia

**Keywords:** EAC, Gambella, High viral load, Ethiopia

## Abstract

**Background:**

Enhanced adherence counseling (EAC) is given to patients with high viral loads (over 1000 copies/ml) to improve Antiretroviral Therapy (ART) adherence, with sessions occurring monthly for three months. A follow-up viral load test is conducted afterwards, but there is a lack of evidence on the impact of EAC on viral load reduction, highlighting the need for further research.

**Objective:**

This study aimed to assess the time to viral load suppression and its predictors among clients receiving EAC at Mattu Karl Comprehensive Specialized Hospital and public hospitals in Gambella town, southwest Ethiopia, 2024.

**Methods:**

A retrospective follow-up study was conducted among 403 randomly selected patients on EAC at Gambella town hospitals and Mettu Karl Comprehensive Specialized Hospital from January 2019 to December 2023. Data were extracted from patient records and analyzed using STATA. Both bivariate and multivariable survival models were used, with a Cox proportional hazard model to identify predictors for viral load suppression. A p-value < 0.05 was considered statistically significant.

**Results:**

The incidence density rate of viral load non-suppression among ART-attending patients was 12.19 (95% CI: 10.9–13.6) per 100 person-months. In the multivariable survival analysis, participants who received Cotrimoxazole Preventive Therapy(CPT) (adjusted HR = 1.55 (95% CI: 1.0–2.3)), disclosed their HIV status (adjusted HR = 1.62 (95% CI: 1.1–2.3)), had fair adherence to ART (adjusted HR = 1.8 (95% CI: 1.1–3.1)), and had no opportunistic infections (adjusted HR = 2.13 (95% CI: 1.3–6.1)) were independent predictors of viral load suppression.

**Conclusion:**

The study found a lower median time to viral load suppression compared to previous Ethiopian studies. Key predictors of suppression included receiving CPT, disclosing HIV status, adhering to ART, and not having opportunistic infections. Emphasis should be placed on providing CPT, encouraging status disclosure, and improving opportunistic infection management.

## Introduction

Globally, AIDS-related illnesses have claimed the lives of over 40.1 million [1] However, the burden of HIV epidemics varies considerably throughout the world with 67% of the global burden concentrated in the African region [2].

In Africa, about 60% of people living with HIV (25.6 million) reside on the continent, which highlights the impact of this disease than any other continent of the world [1]. Since the start of the HIV/AIDS epidemic in Ethiopia, there are a total of 483,127 people living with HIV who are on ART in Ethiopia (national guideline) with viral load coverage and suppression rate of 74.5% and 95% respectively [3, 4]. As a systematic review shows, the Prevalence of virologic failure among HIV patients in sub-Saharan Africa was found to be 17.25% (95% CI 13.39, 21.1) [5] and researches conducted in Ethiopia shows viral suppression after Enhanced Adherence Counseling (EAC) was 51.1% and 66.4% which is below threshold of UNAIDS and WHO targets [6, 7]. The study conducted in some African countries shows that viral load suppression after EAC ranges from 11.8% in Cameroon to 77% in Uganda [8, 9]. Poor adherence to ART is the most common factor that influences viral load suppression [10] and the other one is resistance to current regimen [11]. Many researches were conducted to identify the adherence barriers around the world [12–17].

The main goal of ART drugs is to suppress viral load, but if there are adherence issues it is difficult to achieve viral suppression [3, 18]. A systematic review done from 58 countries shows viral load suppression after EAC is on average 46.1%, and from these papers 48 were found in Africa, and re-suppression is lower in children and adolescents. This review also depicted that among clients with virology failure, nearly 55% only appropriately switched to 2nd line drugs [19].

EAC is a structured intervention recommended by WHO for clients with high viral load. It typically involves three consecutive monthly sessions focusing on Identifying and addressing adherence barriers (such as stigma, forgetfulness, side effects, or lack of support), Providing tailored education on ART, viral load monitoring, and the importance of consistent medication use, Strengthening psychosocial support through case managers, peer supporters, or family involvement, and Monitoring progress with repeat viral load testing after the counseling period [10]. Evidence shows that EAC can lead to significant improvements in viral suppression, with WHO reporting that 70.5% of clients with high viral load suppress after passing through EAC [10].

HIV viral load (VL) suppression is critical in reducing morbidity, mortality, new HIV infections, and drug resistance, and is thus a major strategy in ending HIV/AIDS, especially in sub-Saharan Africa (SSA) [20]. According to recent clinical trial reports, a person maintained with viral load suppression can reduce HIV transmission by 96% [21].

Several significant factors associated with treatment failure and virologic non-suppression among individuals receiving antiretroviral therapy (ART) have been identified. A study found that younger age and higher initial viral load were linked to treatment failure, with the youngest age group exhibiting the lowest adherence and viral suppression rates, while older age groups demonstrated higher rates [16, 17]. Additionally, older age at ART initiation and poor medication adherence were identified as major predictors of virologic non-suppression in pediatric patients [22]. Furthermore, viral load suppression following EAC was lower among individuals on ART for 13–35 months compared to those on ART for fewer than 12 months [23]. Among individuals receiving ART, risk factors for virological failure included an advanced WHO clinical stage and a low CD4 cell count [24]. It is emphasized that high viral load can lead to immunological depletion, increasing susceptibility to opportunistic infection [21].

On the other hand, a high viral load Increases the risk of HIV transmission from mother to child and between discordant couples [25, 26]. In addition the person with high viral load is less productive due to burden of illness and become dependent on family and nation [27]. If a person living with HIV is not virally suppressed, they will have slow immune system recovery, HIV disease advancement, increased morbidity and an enhanced HIV infection transmission risk [28].

Enhanced adherence counseling is recommended by the World Health Organization (WHO) for clients with high viral load, basing on a systematic review that shows 70.5% of clients with high viral load suppress after passing through EAC [18]. Ministry of health Ethiopia (MOH-E) developed national comprehensive HIV prevention, care and treatment guideline recently adapted from WHO also recommends that clients on ART and with high viral load should get EAC for 3 consecutive months; if viral load remains > 1000 copies/mL despite good adherence, and virological failure is confirmed, they meet criteria to switch to the next-line regimen.

in addition to this guide line also recommended test and treat strategy, routine and target viral load measures that is gold standard for early detection of treatment failure (monitoring and diagnosis) purpose [3, 29].There are adherence case managers and adherence supporters hired at high case load facilities to support clients with high viral load by identifying and helping with adherence barriers during EAC sessions [30]. Drug optimization and availability of combination of drugs are some of the interventions tried to reach UNAIDS target on time [29].

Studies conducted in Ethiopia and other regions show variations in the time to viral suppression, with durations ranging from 90 to 181 days in studies at Arbaminch General Hospital and East Shewa Zone [31, 32]. The predictors of time to viral suppression are not yet well understood in these settings. Therefore, this study aims to estimate the time to viral suppression and identify predictors of viral load suppression among adult HIV patients on ART in Southwest Ethiopia, specifically at Gambella General Hospital, Gambella Primary Hospital, and Mettu Karl Comprehensive Specialized Hospital.

## Methods

### Study area and period

Gambella Region, located about 670 km southwest of Addis Ababa, borders South Sudan, Oromia, and The Southwest Ethiopia Peoples Region. With an estimated population of 483,097, nearly 75% live in rural areas. Gambella Hospital and Gambella General Hospital offer specialized HIV care to communities, including refugees. Mettu Karl Comprehensive Specialized Hospital located 600 km from Addis Ababa in Mettu town serves over 1.4 million people across Oromia, Gambella, and The Southwest Ethiopia Peoples’ Region, providing advanced HIV prevention, treatment, and referral services. The study was conducted at both hospitals from June 2024 to September 2024.

### Study design, population, and sample size

An institution-based retrospective follow-up study was conducted among clients with high viral loads from January 2019 to December 2023. Sample size was determined via factors significantly associated with viral load suppression from previous studies [33] and was calculated via the Schoenfeld formula via Stata software version 14.

$$\begin{aligned}&E=\frac{{\left(\frac{Z\alpha\:}{2}+Z\beta\:\right)}^{2\:\:}}{P1P2{\left(lnHR\right)}^{2\:\:}}\cr & \mathrm{And}\:n=\frac{E}{P\left(E\right)}\:=\mathrm{Schoenfeld}\:\mathrm{formula}\end{aligned}$$

Sample size was estimated using Schoenfeld’s formula for survival analysis, based on hazard ratios and event probabilities as shown in Table 1.

**Table 1:** Sample Size determination based on Schoenfeld’s formula using hazard ratios and event probabilities

Assumption	Variables	HR	Probability of event	Sample size
Type I error = 0.05 power = 80% withdrawal probability = 0.1	Tuberculosis preventive therapy	2.24	0.861	366
	Baseline WHO clinical stage	2.12	0.861	288

HR = Hazard Ratio

Therefore, the maximum sample size is taken, which is 366, and 10% contingency added = 403 [33].

### Eligibility criteria

#### Inclusion criteria

All records of clients on Highly Active Antiretroviral Therapy (HAART) with initial high viral loads who received EAC at Gambella town hospitals and Mattu Karl Comprehensive Specialized Hospital (MKCSH) from January 2019 to December 2023 were obtained.

#### Exclusion criteria

Clients were excluded if either baseline CD4 count or date of first viral load measurement was missing from their records between January 2019 and January 2023.

### Sampling procedure

**Fig. 1:**
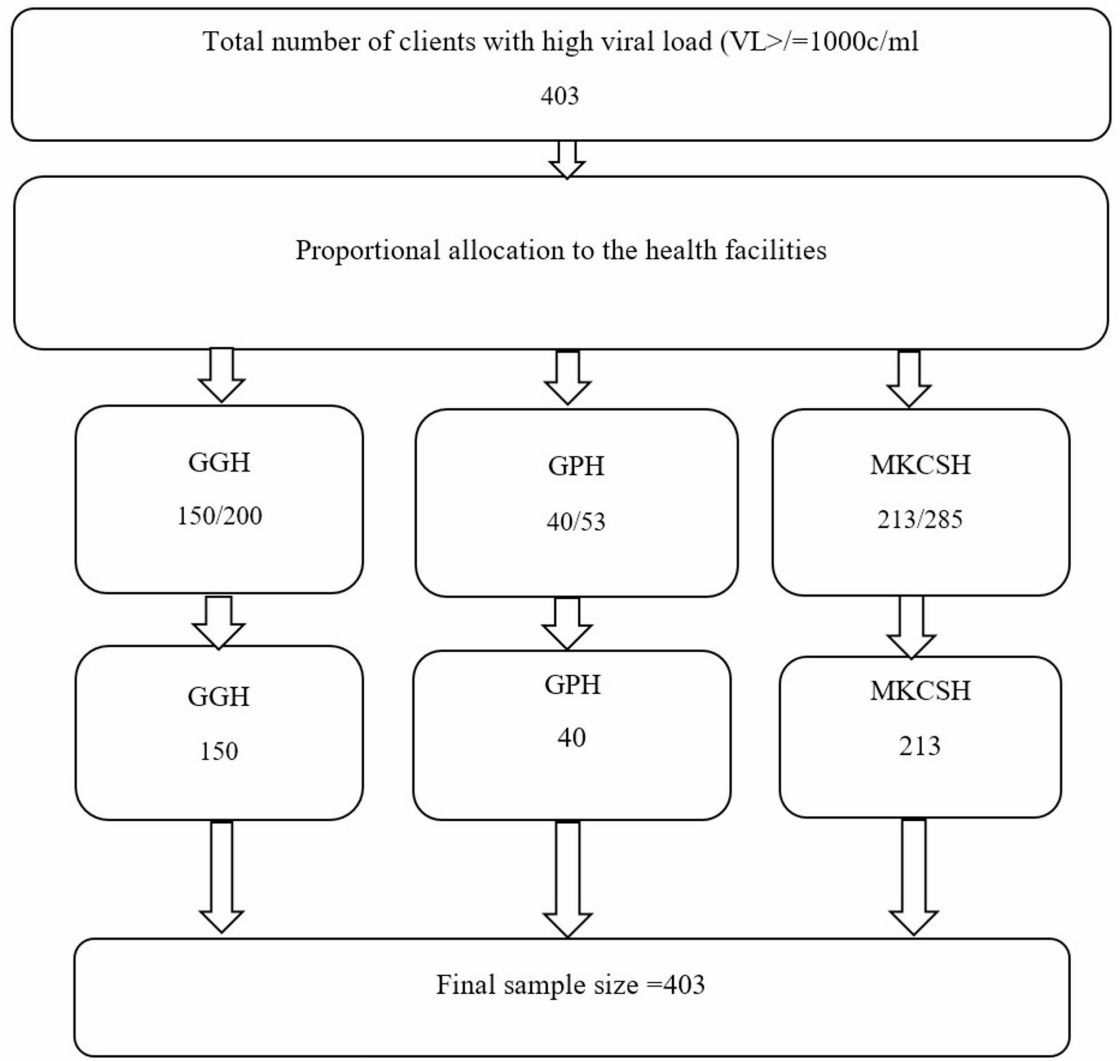
Schematic representation of sampling

The number of patients followed up differed across hospitals: 53 at Gambella Town Primary Hospital (GPH), 285 at Mettu Karl Comprehensive Specialized Hospital (MKCSH), and 200 at Gambella General Hospital (GGH). To ensure proportional representation, 40 patients (75.5%) were included from GPH, 213 patients (74.7%) from MKCSH, and 150 patients (75%) from GGH, giving a final sample size of 403. This distribution is illustrated in Fig. 1.

**Fig. 2:**
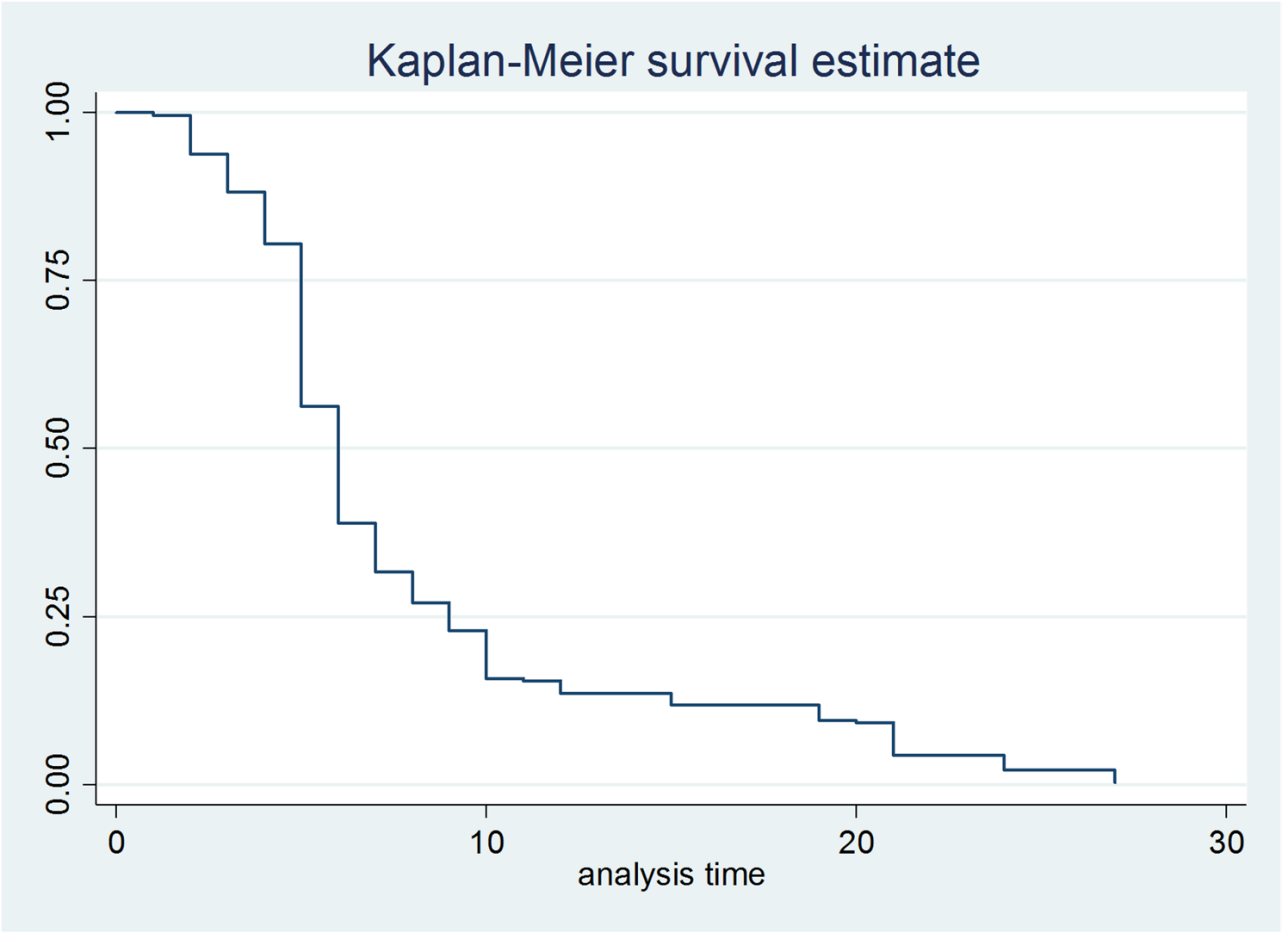
Kaplan–Meier estimate of viral load suppression among patients attending ART at MKCSH, GHH and GPH, 2024

**Fig. 3:**
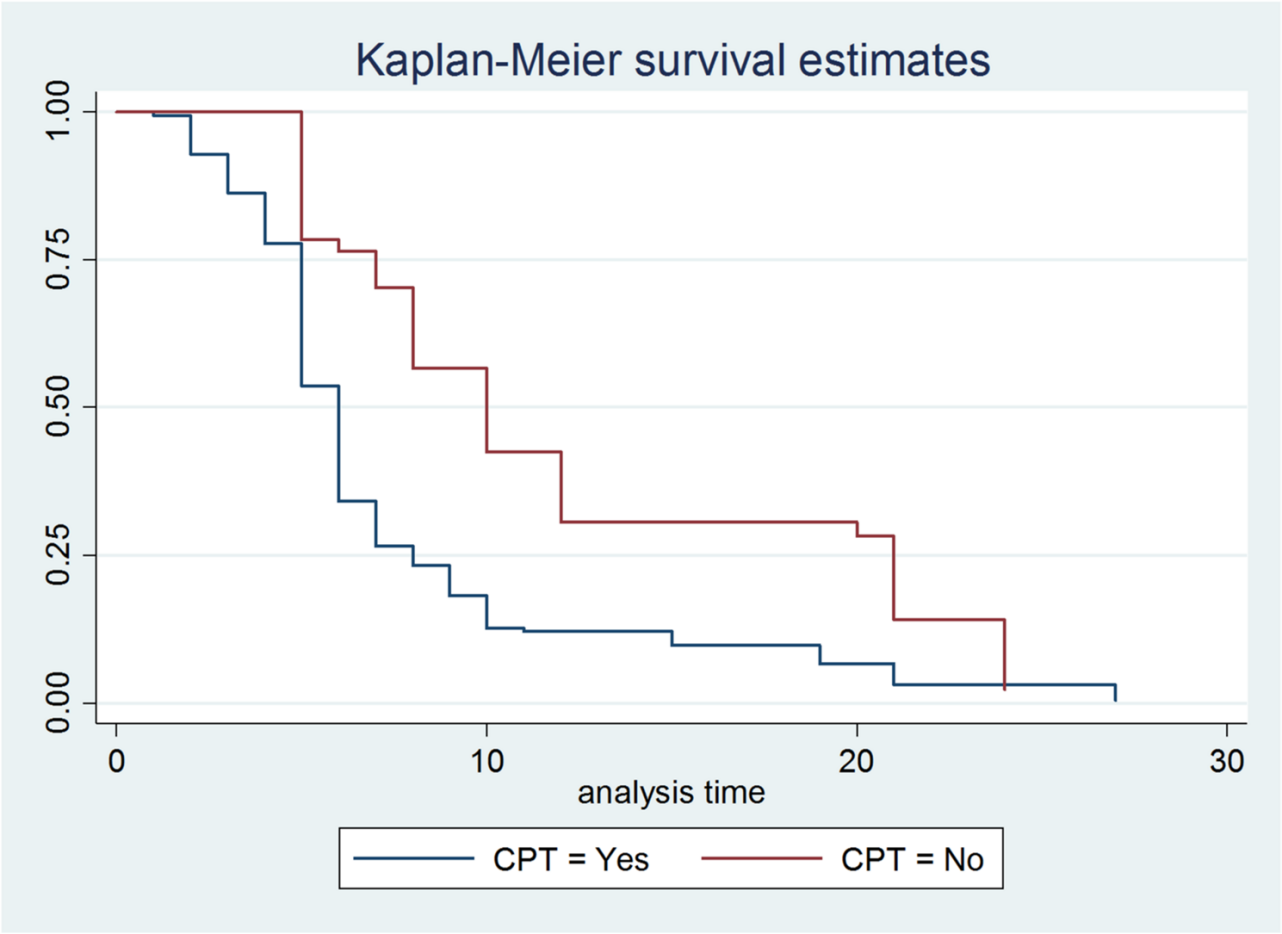
Kaplan–Meier survival estimate by CPT initiation among patients attending ART at selected Hospitals in Gambela town and MKCSH Southwest thiopia, 2024

Patients with high viral loads during the study period were identified from laboratory high-viral-load registration logbooks. The medical numbers of all clients with high viral loads were extracted from electronic medical registration or laboratory viral load registration logbooks. Subsequently, clients whose first viral load results were archived in their medical follow-up charts were selected for data collection. The clients with achieved repeated viral load results were line-listed and selected for the study unit via a systematic sampling method. This systematic approach ensured comprehensive data collection and representation from all health facilities.

Accordingly, all charts containing detailed information about patients who were on ART were reviewed. If incomplete data were encountered, the data collectors tried to obtain information from different data sources (patient charts and follow-up forms). Additionally, if clinical parameters and laboratory results (CD4 count and WHO clinical stage) were not found at the start of EAC sessions, the data that were most recent to the starting date of EAC sessions were considered baseline data. Behavioral parameters, such as disclosure, family support and substance abuse, were collected from the client’s high viral load follow-up form, which was assessed during enrollment to EAC and then after sociodemographic data were taken from electronic medical record because they are frequently updated. Client-related factors such as Lost to Follow-Up (LTFUP), missed ARV (antiretroviral) and the use of medications other than ART were collected from client follow-up charts. The time between HVL (High Viral Load) detection and EAC initiation and the duration (in months) of EAC and TAT (Turnaround Time) were collected from the HVL follow-up form and client follow-up chart and calculated accordingly. Three nurses, as data collectors, and two ART focal persons, as supervisors, participated in the data collection process.

### Operational definitions

#### Event

Viral load suppression.

#### Censored

ART patients who died before the second VL test status, were transferred out before the second VL test status, were lost from follow-up before the second VL test status, and failed to suppress until the end of follow-up.

#### Time to viral load suppression

Time to viral load suppression was defined as the duration from enrollment into EAC to the first viral load < 1000 copies/ml, among clients who had an initial high viral load.

#### Transfer in

If a diagnosis was made outside the study sites.

#### Transfer out

Move by taking the full medical record to other health institutions for care and treatment.

#### Opportunistic infections

Illnesses caused by various organisms, which usually cause disease in individuals with a weakened immune system. It is classified as yes or no.

#### Functional status

Focus on the patient’s ability to perform basic activities of daily living, which include basic self-care, such as feeding, toileting, cooking, shopping, bathing, and managing one’s affairs. It was classified as working/ambulatory and bedridden.

#### Adherence level

The percentage of ARV drugs taken, based on self-reported pill counts, was used as a criterion to classify drug adherence level (good is defined as ≥ 95% of doses taken as prescribed, fair is defined as 85–94% of doses taken, and poor is defined as < 85% of doses taken).

#### Baseline CD4 counts

This is the CD4 count at the initiation of ART. It is classified as < 200 cells/mm3 or ≥ 200 cells/mm3.

**Viral load suppression**: A viral load of < 1000 RNA copies/mL after first-line ART HIV-positive adults: patients on ART who are older than 15 years.

#### WHO clinical stage

This is an indicator of patient status and is classified as I, II, III or IV depending on some clinical parameters. In this study, we classified the cases as I or II and III or IV.

#### Incidence density of VL suppression

number of cases that experienced VL suppression during the follow-up period per sum of the lengths of time each study participant was observed and at risk of experiencing VL suppression.

#### Patient-months of observations

the sum of the lengths of time each study participant was observed and at risk of experiencing VL suppression.

### Data quality assurance

Data quality was assured through the design of a structured data collection tool, which consisted of a standardized checklist and extraction form developed based on national HIV guidelines and relevant literature. This tool ensured that all required variables were consistently captured across sites, reducing errors and omissions. Training was given to both the data collectors and supervisors on the objectives of the study, data extraction procedures, recording of patient charts, and registration of books using the prepared checklist. Pretesting was performed on 5% of the overall sample at the Abobo Catholic Health Center. Following the pretest, the tool was refined variables such as spouse HIV status were added, while income and smoking status were removed to improve relevance and clarity. During the data collection period, a supervisor was assigned to monitor completeness and accuracy, ensuring no missing data. The overall activities of data collection were closely supervised and controlled by the principal investigator, thereby strengthening quality assurance throughout the process.

### Data processing and analysis

The data were entered into EpiData version 3.1 [34] and exported to STATA 14.1 for analysis [35]. The data were then cleaned by observing the frequency, cross tabulation tables, and sorting to check for missed values and outliers. Descriptive statistics, including the means, medians, standard deviations and frequency tables, were used to describe the characteristics of the study participants for continuous variables with normal or nonnormally distributed data and categorical data.

The probability of viral load suppression was estimated via the Kaplan‒Meier (KM) method. KM was again used to compare survival time between groups of categorical variables. Variables with p values less than 0.2 in the bivariable analysis were included in the multivariable survival models. The Cox proportional hazard (PH) assumption was checked via Schoenfeld residuals, and the Cox-Snell residual test was used to check the adequacy of the final model. A multivariate Cox proportional hazard regression model was then fitted. Finally, variables with *P* < .05 in the multivariable analysis were considered predictors of time to viral load suppression.

## Results

### Sociodemographic characteristics of high-viral-load individuals

Data were obtained through a retrospective review of patient medical records. A total of 388 patient files met the inclusion criteria and were analyzed. Based on the available documentation, the dataset represented 96.3% of all eligible patient charts during the study period. The mean age of the patients was 32.7 years (SD ± 10.8), with 168 males (43.3%) and 220 females (56.7%). Most participants, 356 (91.8%), were from urban areas, whereas 32 (8.3%) resided in rural regions. In terms of religious affiliation, 217 participants (55.9%) identified as Orthodox, 51 (13.1%) as Muslim, 104 (26.8%) as Protestant, and 16 (4.1%) as Catholic. This sample shows a predominantly urban population with a majority following Orthodox faith, while females were slightly more represented than males. The majority of participants had primary education (43.3%), whereas the minority were unable to read and write (5.7%).

### Baseline clinical and laboratory-related characteristics

The study revealed that the majority were working (74.5%), with no history of opportunistic infections (82.7%) or hospital admissions (83%). Most participants were in WHO Stage II (58.2%), with baseline CD4 counts between 201 and 349 cells/mm³ (63.9%) and viral loads below 5000 copies/mL (45.9%) (Table 2).

**Table 2:** Socio-demographic, clinical and laboratory characteristics after enhanced adherence counseling intervention among people living with HIV during follow-up at public hospitals in Gambella town and MKCSH southwest Ethiopia in 2024. (*N* = 388)

Variables	Category	Survival statues		Total
		VL suppressed	Censored	
Age category	< 18	74	11	85
	18–29	124	20	144
	30–39	97	14	111
	> 40	41	7	48
Sex	Male	146	22	168
	Female	190	30	220
Residency	Urban	311	45	356
	Rural	25	7	32
Religion	Orthodox	191	26	217
	Muslim	44	7	51
	Protestant	87	17	104
	Catholic	14	2	16
Marital status	Never married	92	11	103
	Married	188	31	219
	Divorced	32	8	40
	Widowed	24	2	26
education level	Cannot read and write	18	4	22
	Primary	189	26	215
	Secondary	77	13	90
	Collage and above	52	9	61
Functional status	Working	255	34	289
	Ambulatory	49	12	61
	Bedridden	32	6	38
Presence of OI	yes	8	59	67
	no	272	49	321
History of admission	No	279	43	322
	Yes	57	9	66
WHO clinical stage	Stage I	119	22	141
	Stage II	205	21	226
	Stage III	12	9	21
Spouse HIV status	Positive	266	31	297
	Negative	22	69	91
Baseline CD4 count	< 200	6	9	15
	201–349	217	31	248
	> 350	113	12	125
Baseline VL count	< 5000	114	64	178
	5000–49,999	97	36	133
	> 50,000	63	14	77

VL−viral load OI − Opportunistic infection

### Behavioral and social characteristics

The study revealed that the majority of participants disclosed their HIV status (305, 78.6%) and did not receive family support (316, 81.4%). Most participants were confident in taking ART (206, 53.1%) and did not consistently use condoms (282, 73.2%). A larger portion did not experience hopelessness (212, 54.6%) or loss of interest (222, 57.2%). Substance use was reported by 127 participants (32.7%), and 170 (43.8%) faced food shortages. ART discontinuation was reported by 207 (53.4%) participants, whereas most participants (348, 89.7%) attended all enhanced adherence counseling (EAC) sessions (Table 3).

**Table 3:** Behavioral and social characteristics after enhanced adherence counseling intervention among people living with HIV during follow-up at public hospitals in Gambella town and MKCSH southwest Ethiopia 2024 (*N* = 388)

Variables	Category	Survival statues		Total
		VL suppressed	Censored	
HIV disclosure status	No	72	11	83
	Yes	264	41	305
Family support	No	273	43	316
	Yes	63	9	72
Confident to take ART	No	158	24	182
	Yes	178	28	206
Condom use	No	239	43	282
	Yes	97	9	106
Feeling hopelessness	No	182	30	212
	Yes	154	22	176
Loss of interest	No	143	23	222
	Yes	193	29	166
Substance use	No	229	32	261
	Yes	107	20	127
Food shortage	No	183	35	218
	Yes	153	17	170
ART discontinuation	No	155	22	177
	Yes	177	30	207
Attending all EAC	No	32	8	40
	Yes	304	44	348

### Treatment-related characteristics (Table 4)

**Table 4:** ART and treatment-related characteristics after enhanced adherence counseling intervention among people living with HIV during follow-up at public hospitals in Gambella town and MKCSH southwest Ethiopia 2024. (*N* = 388)

Variables	Category	Survival statues	
		VL suppressed	Censored
Duration of ART	≤ 59 month	35	14
	> 59 month	234	97
Interrupted follow-up	No	257	40
	Yes	79	12
CPT treatment	No	46	6
	Yes	290	46
INH treatment	No	217	30
	Yes	119	22
ARV dose	Once	297	47
	Twice	39	5
ART regimen	1st line	309	47
	2nd line	25	5
	3rd line	2	0
Missed ARV dose	No	186	29
	Yes	150	23
Number of dose miss 1daily regimen	< 2 dose	39	6
	2–4	68	9
	≥ 5	31	7
Number of dose miss 2 daily regimens	≤ 3 dose	23	2
	4–9 dose	25	4
	> 9	11	1
Missed dose since started ARV	No	270	43
	Yes	64	9
Adherence	Good	244	20
	Fair Poor	77 6	25 16

ART − Antiretroviral Therapy CPT − Cotrimoxazole Preventive Therapy INH – Isoniazid ARV−antiretroviral

### Incidence of viral load suppression

The study participants were followed for a total of 2,758 months, with a median of six months. The incidence density rate of non-suppression of the viral load among ART patients was 12.19 (95% CI: 10.9–13.6) per 100 person-months.

### Kaplan–Meier estimates of the time to viral load suppression

The overall median viral load suppression time in this study was 6 months (95% CI: 8.22, 9.78) (Fig. 2).

The Kaplan–Meier analysis highlights important factors affecting viral load suppression among the study participants. Patients who received Cotrimoxazole Preventive Therapy (CPT) had a shorter duration of viral load suppression, indicating the effectiveness of CPT in promoting viral load suppression (Fig. 3). 

**Fig. 4:**
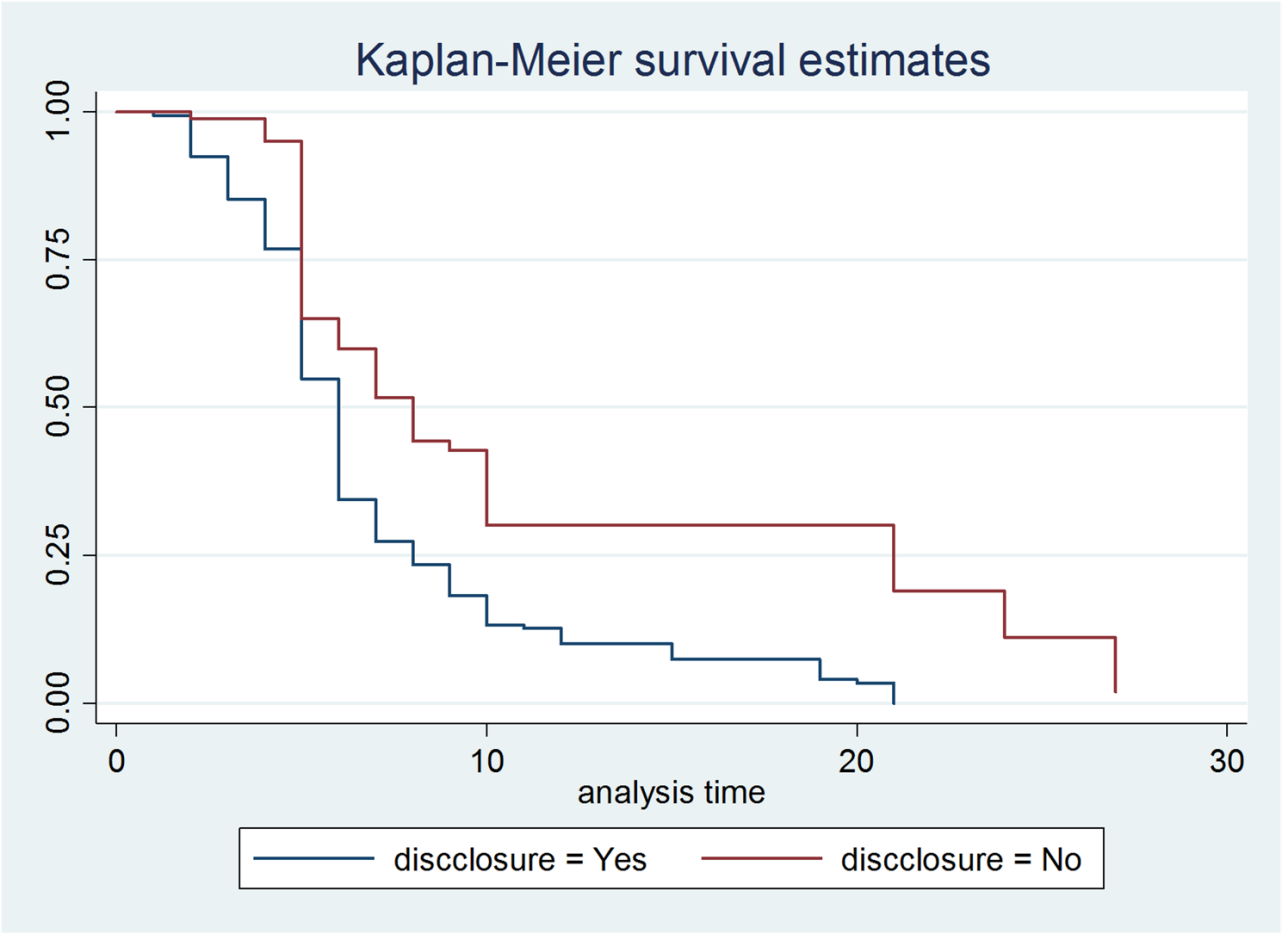
Kaplan–Meier survival estimate by disclosure status among patients attending ART at selected Hospitals in Gambela town and MKCSH Southwest thiopia, 2024

Furthermore, patients who disclosed their HIV status to their partners had faster suppression times than those who did not (Fig. 4).

### Testing overall model fit

**Fig. 5:**
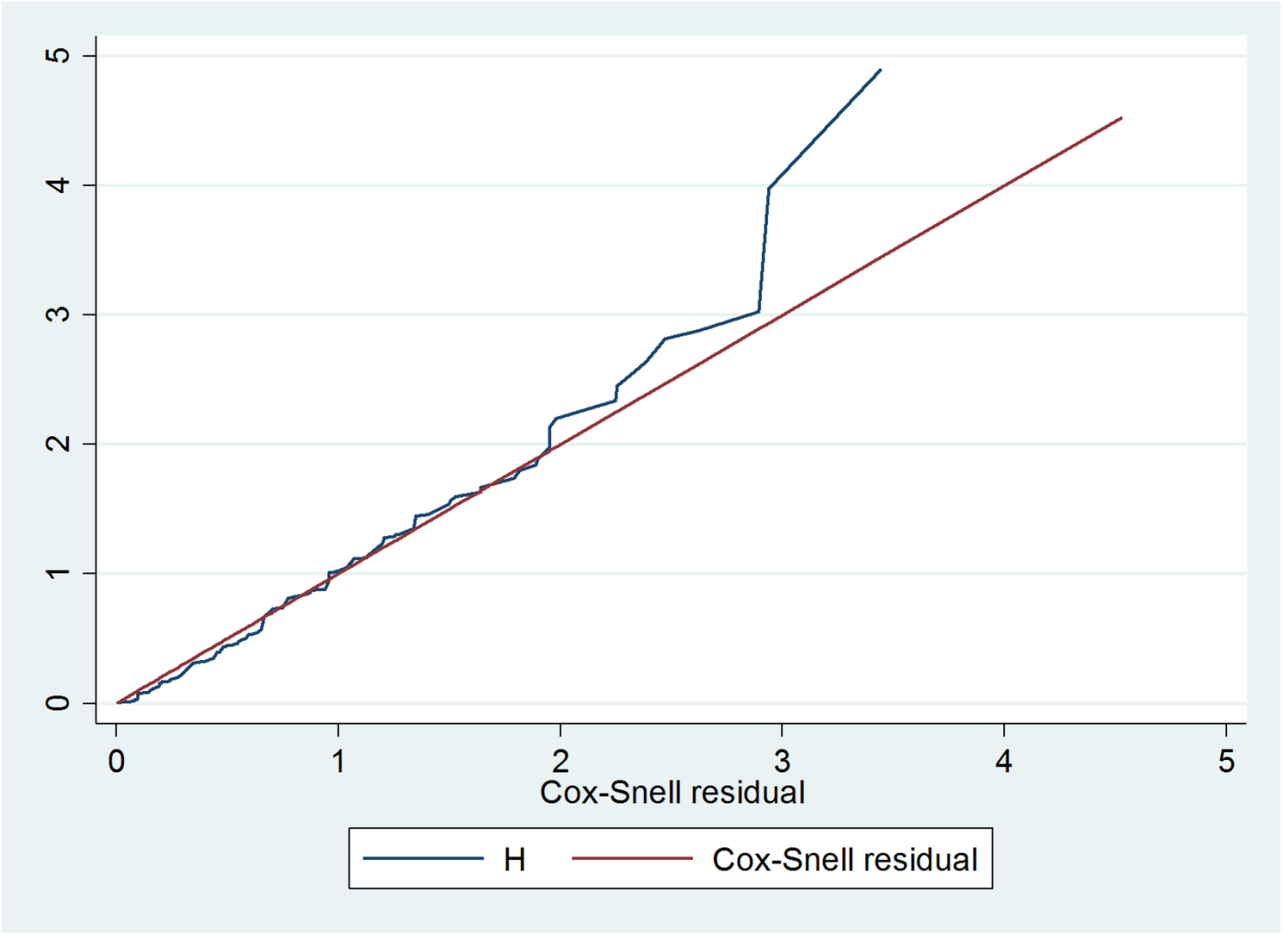
Cox-Snell residual graph on loss to follow-up among clients attending ART at MKCSH, GHH and GPH, 2024

**Global test of proportional hazards assumption** The proportional hazard assumption was satisfied on the basis of the global test (p-value = 0.464). Assessment of the adequacy of the Cox regression model: The adequacy of a final fitted model was assessed with the Cox–Snell residual. The plot of the cumulative hazard function of the Cox–Snell residuals against maximum likelihood estimation with cumulative hazard functions is presented in Fig 5. The plot shows that the cumulative hazard function of the residuals against the ox-Snell residuals was approximately a straight line; hence, the model fit the data (Fig. 5).

### Predictors of viral load suppression

In the bivariable survival analysis, several factors were examined as candidate variables for the multivariable survival analysis. This included isoniazid (INH) use, interrupted follow-up, CD4 count, drug adherence, disclosure status, CPT, family support, WHO clinical stage, and the presence of opportunistic infections (OIs). Each of these variables played a role in influencing the outcomes of VS, with varying degrees of significance observed across the study participants. However, in the multivariable survival analysis, drug adherence, disclosure status, CPT, and the presence of OIs were independent predictors. Participants receiving CPT had a significantly shorter time to achieve viral load suppression over time (adjusted HR = 1.55, 95% CI: 1.0–2.3). Those who disclosed their HIV status were more likely to have a shorter duration of viral suppression (adjusted HR = 1.62, 95% CI: 1.1–2.3). Similarly, fair adherence to ART increased the likelihood of faster viral suppression than poor adherence did (adjusted HR = 1.8, 95% CI: 1.1–3.1). Additionally, those without opportunistic infections achieved quicker viral load suppression (adjusted HR = 2.13, 95% CI: 1.3–6.1) (Table 5).

**Table 5:** Multivariable analysis of predictors of viral load suppression after enhanced adherence counseling intervention among people living with HIV during follow-up at public hospitals in Gambella town and MKCSH in southwestern Ethiopia in 2024. (*N* = 388)

Covariates	Category	Survival status		Crude Hazard ratio (95% CI)	Adjusted Hazard ratio (95% CI)
		VL suppressed	Censored		
INH	No	217	30	1	1
	Yes	119	22	1.7 (1.4–2.2)	1.1 (0.8–1.5)
Interrupted follow-up	No	257	40	0.95 (0.7–1.2)	1.0 (0.7–1.3)
	Yes	79	12	1	1
CD4 count	< 200	6	9	1	1
	201–349	217	31	1.0 (0.4–2.2)	0.9 (0.4–2.3)
	> 350	113	12	1.1 (0.9–1.5)	1.1 (0.8–1.5)
Drug adherence	Good	244	20	1.56(0.0-2.5)	1.2(0.7–2.1)
	Fair	77	25	**1.89(0.5–1.4)**	1.8(1.1–3.1) **
	Poor	6	16	1	1
Disclosure status	No	72	11	1	1
	Yes	264	41	**2.05 (1.5–2.7)**	1.62 (1.1–2.3) **
CPT	No	46	6	1	1
	Yes	290	46	**1.9 (1.4–2.6)**	1.55 (1.0-2.3) *
Family support	No	273	43	1	1
	Yes	63	9	1.13 (0.8–1.4)	1.17(0.8–1.6)
WHO clinical stage	Stage I	119	22	1	1
	Stage II	205	21	**1.04 (0.5–1.9)**	**1.01 (0.7–2.6)**
	Stage III	12	9	1.38(0.7–2.4)	1.6(1.1–2.3)
OI	No	272	49	**2.24 (1.1–4.9)**	2.13 (1.3–6.1) **
	Yes	8	59	1	1

OI − Opportunistic infection CPT − Cotrimoxazole preventive therapy INH – Isoniazid CI = Confidence Interval *p* < .05= *, *p* < .01= **

## Discussion

This study assessed the incidence rate of viral load suppression after EAC and its predictors among HIV-positive adults in Southwest Ethiopia. This study revealed that the incidence density rate of viral load non-suppression was 12.19 per 100 person-months (95% CI: 10.9–13.6), which is comparable with that reported in a study performed in the West Gojam Zone, which reported a viral load suppression rate of 11.17 per 100 person-months [36]. The similarity could be due to the same methodology. This study revealed a longer median time to viral suppression than did the study conducted at Arba Minch General Hospital, which reported a median time to viral load suppression of 3 months [31]. This may be due to factors such as sample size differences, differences in ART adherence or patient health status.

Compared to the retrospective study in Hosanna, which reported a viral load suppression incidence rate of 9.68 per 100 person-months and a median suppression time of 9 months, our study showed a higher suppression rate (12.19 per 100 person-months) and a shorter median time (6 months) [33].

This study revealed a shorter median time to suppression (6 months) than the 9 months reported in the Hosanna study did, indicating that viral load suppression occurred more rapidly here despite the higher incidence rate. These differences may be attributed to factors such as regional variations in ART management, patient health status, or adherence practices.

This study revealed that participants receiving CPT had a shorter duration of viral load suppression. This finding is similar to that of a study at Arba Mich Hospital [31]. This finding can be attributed to the role of CPT in preventing opportunistic infections (OIs) and supporting the immune system. CPT is commonly used in HIV-positive individuals to reduce the risk of bacterial infections, particularly those that can further weaken the immune system. This implies that HIV care programs should adopt a holistic approach that includes both ART and preventive therapies such as CPT.

The study further revealed that those who disclosed their HIV status to their partners were more likely to have shorter viral suppression sooner. This could be attributed to the fact that disclosure often leads to increased emotional support, understanding, and encouragement from partners, which may enhance treatment adherence and reduce stress [37]. Furthermore, when a partner is aware of the individual’s HIV status, they may be more likely to support the patient in seeking medical care, attending follow-up appointments, and adhering to antiretroviral therapy (ART). This finding is in line with the findings of a systematic review from Ethiopia [38]. This finding implies that HIV care programs could consider offering counseling and support to patients to facilitate safe and supportive disclosure of their HIV status to their partners.

The study revealed that fair ART adherence was associated with shorter viral suppression. This could be because fair adherence allows for a substantial reduction in the viral load and a better response to treatment. This finding is consistent with a study conducted at Arba Minch General Hospital, where adherence to ART was also shown to positively influence viral suppression [31]. And a systematic review in Ethiopia [38]. This finding implies that HIV treatment programs should prioritize improving adherence, even at moderate levels. Interventions such as adherence counseling, reminders, and support groups can be implemented to help patients maintain regular ART use and achieve better outcomes.

The study revealed that those without opportunistic infections achieved viral suppression more quickly. The association between the absence of opportunistic infections (OIs) and quicker viral suppression can be attributed to the fact that OIs often contribute to immune system weakening, which may reduce the body’s ability to effectively control viral replication. Individuals without OIs are likely to have a more robust immune response, allowing them to better manage their HIV infection and respond more effectively to antiretroviral therapy (ART). This finding aligns with that of a study in Ethiopia [13, 36, 38]. This finding implies that programs should prioritize the prevention, early diagnosis, and management of opportunistic infections in HIV-positive patients.

### Strength and limitations of the study

The study’s strengths include its inclusion of all patients with high viral loads over the past five years, and its retrospective, institution-based follow-up design, which utilized primary secondary data sources such as patient charts, follow-up records, and high viral load registration logs, all of which are key documentation for patient information in the country. However, the study also had limitations, as the analysis was confined to the variables available in these data sources. Additionally, important potential predictor variables, such as spouse education status, wealth index, smoking status, and clients’ lived experiences, were not considered due to their absence in the available data.

## Conclusion

The overall viral load suppression observed in this study was suboptimal compared to global targets and national expectations. Factors such as CPT, HIV status disclosure, absence of opportunistic infections, and fair adherence to ART were associated with faster viral suppression. Stakeholders should strengthen adherence support and preventive care strategies to accelerate viral suppression and improve treatment outcomes.

## Data Availability

Data will be available upon the request of the corresponding author.

## Abbreviations

AIDS: Acquired Immunodeficiency Syndrome
ART: Antiretroviral Therapy
CPT: Cotrimoxazole Preventive Therapy
EAC: Enhanced Adherence Counseling
HIV: Human Immunodeficiency Virus
INH: Isoniazid
MKCSH: Mattu Karl Comprehensive Specialized Hospital
MOA: Months on Antiretroviral Therapy
MoH-E: Ministry of Health – Ethiopia
OI: Opportunistic Infection
TAT: Turnaround Time
UNAIDS: Joint United Nations Programme on HIV/AIDS
VLS: Viral Load Suppression
WHO: World Health Organization
