# Correction: Time to viral load suppression after enhanced adherence counseling among HIV patients attending antiretroviral therapy in Southwest Ethiopia

**DOI:** 10.1186/s12879-026-13904-4

**Published:** 2026-07-06

**Authors:** Haymanot Berihun, Abel Felege, Daniel Girma, Dereje Tsegaye

**Affiliations:** Department of Public Health, College of Health Sciences, Mattu University, Mattu, Ethiopia

**Correction: BMC Infect Dis. 2026;26:676**

10.1186/s12879-026-12957-9

In this article, the author Dereje Tsegaye was inadvertently omitted from the author list. The correct author group should be:

Haymanot Berihun^1*^, Abel Felege^1^, Daniel Girma^1^, and Dereje Tsegaye^1^.

The original article has been corrected.

